# A Serious Game for Cognitive Stimulation of Older People With Mild Cognitive Impairment: Design and Pilot Usability Study

**DOI:** 10.2196/41437

**Published:** 2024-04-04

**Authors:** Juan Francisco Ortega Morán, J Blas Pagador, Vicente Gilete Preciado, José Luis Moyano-Cuevas, Trinidad Rodríguez Domínguez, Marta Santurino Muñoz, Francisco M Sánchez Margallo

**Affiliations:** 1 Centro de Cirugía de Mínima Invasión Jesús Usón, Cáceres, Spain; 2 Robolab, FENTO, Universidad de Extremadura, Cáceres, Spain

**Keywords:** serious game, mild cognitive impairment, cognitive stimulation, design, pilot study, older people, activities of daily living, shopping, ecological validity

## Abstract

**Background:**

Cognitive stimulation of older people helps prevent, and even treat, age-related diseases, such as mild cognitive impairment. Playing games reduces the probability of experiencing this pathology, which is related to the loss of the ability to carry out some instrumental activities of daily living.

**Objective:**

This work describes the design and development of a serious game for the cognitive stimulation of older people, with exercises related to the daily life task of shopping. A pilot study for its preliminary usability validation is also presented.

**Methods:**

The designed serious game includes 4 exercises consisting of shopping in a hypermarket, ordering products, making payments, and organizing the purchase, thus dealing with the most frequent cognitive problems of older people associated with episodic declarative memory, naming, calculation, and organization, respectively.

**Results:**

A total of 19 older people participated in the pilot study for the usability validation of the serious game. They indicated that they like the aesthetic and interesting topic of the game. They reported that it provides a high level of entertainment and could be useful in daily life for mental stimulation. The participants found the serious game to be intuitive, but the ease of use and readability of the instructions could be improved.

**Conclusions:**

This study suggests that the innovative serious game developed could be accepted by older people for their cognitive stimulation to prevent or treat mild cognitive impairment, although a long-term intervention study should be performed as future work. Its ecological validity design, with everyday tasks, adaptable levels of difficulty, and motivational mechanisms, is a differentiating factor compared to similar serious games.

## Introduction

The increase in life expectancy means that we can live with age-related pathologies, such as dementia. However, the prevalence of this pathology is clearly on the rise, and there are currently about 55 million people affected worldwide [1] and around 11 million for the broad European region [2]. This acquired syndrome is characterized by a progressive deterioration of cognitive functions, which influences activities of daily living (ADL) and decreases the level of independence of individuals. For this reason, dementia is classified into grades, where simple activities can be performed in the mild stage but the severity of symptoms increases as the impairment progresses [2-4]. Mild cognitive impairment (MCI) is the term used for individuals whose cognitive changes fall between those of aging and early dementia and is considered a precursor to early dementia in around 30% of cases; thus, those with MCI are much more likely to progress to this type or level of dementia, especially in older people [4]. Treatment usually includes drugs that aim to reduce or delay cognitive, psychological, and behavioral symptoms [5], but nonpharmacological treatments are also very important. These include reality orientation, reminiscence and validation therapies, or cognitive stimulation [6].

Cognitive stimulation includes techniques that focus on treating cognitive aspects through activities that allow them to be worked on globally and simultaneously [6,7]. Cognitive and reminiscence activities, multisensory activities, as well as those that work on social aspects are used, along with group activities that facilitate integration and social participation among users [8]. In fact, several studies confirm an improvement in different aspects of the cognitive sphere and in the quality of life of people with dementia who have participated in this type of therapies [9,10]. We also found improvements in mood and ADL maintenance [11].

Currently, information and communication technology (ICT) instruments and tools are incorporated into nonpharmacological treatments. These technological devices facilitate performance, which can lead to a greater sense of self-efficacy and improve one’s own perception of functionality, as well as reduce the burden of caregivers. Some of the devices that are commonly used with people with dementia are tele-assistance, devices that improve cognitive functions, and robotics [12]. Among them, the most widely used are those that can be connected to the internet (tablets, mobile phones, computers, and video game consoles), which provides a wide variety of resources that allow the diversification of therapies and increase motivation and adherence to treatment [8,13]. The use of ICTs as therapeutic tools requires a previous study of the person’s abilities and skills for their use and management, to avoid feelings of frustration that can lead to discouragement from therapy or rejection of the device [9]. Therefore, it is essential to gradually approach the device and its applications, looking for intuitive tasks that the user can easily carry out independently [9,13].

Serious games are ICT games whose main objective is to give a therapeutic or diagnosis value to the playful action of the games. They have been tested in different areas of intervention in pathologies with cognitive impairment [14,15], particularly dementia [16]. Through serious games, we can work to delay deficits, increase autonomy and relationships with their social environment, and improve the quality of life of people with dementia [17]. For this intervention, the occupational therapist may include in their individual intervention plans games to work on physical, cognitive, and social aspects. The literature reveals that there are already a number of well-established serious games that could improve older people’s cognitive health. Some examples of these are focused on sport simulations, quizzes with text and images, music tools, arithmetic and reading calculations, etc [17]. Others instead use a fantasy world setting, minigames, puzzles, or scenarios to cope with stressful or negative situations [18]. However, few serious games have been developed that incorporate tasks focused on ADL, such as cooking [19]. Once cognitive impairment becomes perceptible in ADL, the disease has usually progressed, so the analysis of ADL performance would help to assess cognitive status during the course of cognitive impairment [19]. In this sense, serious games can be a valuable tool for achieving this in a motivating and enjoyable way for older people with ecological scenarios.

In this study, we describe the design and development process of a serious game developed for the cognitive stimulation of older people, with exercises related to the daily life task of shopping. We also present the results of a usability validation from a pilot study of the game.

## Methods

### Game Development Process and Design Requirements

In the process of designing and developing this serious game, researchers with a bioengineering profile, game designers and developers (computer and telecommunication engineers), and health care professionals (neuropsychologists, psychologists, and occupational therapists) participated to fulfill the essential collaboration of an interdisciplinary team [20].

First, 2 focus groups were performed with 11 psychologists and 6 occupational therapists from Extremadura, Spain, to identify the needs, limitations, and motivations of older people to use cognitive stimulation programs. The selection criteria of participants in both focus groups were the geographical location (working in rural and urban areas) and the kind of institutions in which they work (public and private). The focus groups were conducted by 2 engineers with extensive experience in conducting interviews. After the presentation of the study and the objectives, a previously created guide with topics of interest and questions was followed to conduct the session in a semistructured way. With the participants’ consent, all conversations were recorded for subsequent transcription and analysis. NVivo 2017 (QSR International) software was used to carry out this analysis to facilitate the drawing of conclusions. Four thematic areas were identified: (1) the most frequent cognitive problems of older people are mainly focused on memory loss, disorientation, difficulty in performing executive tasks, or difficulty in concentrating; (2) motivation is the fundamental element for the success of new training exercises; (3) technological barriers are mainly related to interface design problems and cultural level; and (4) the low degree of awareness of older people regarding the importance of leading an active life. This served as a basis for designing the new tool to promote the cognitive training that directly stimulates memory and executive tasks, as well as orientation and concentration skills.

In the implementation of this serious game for Android OS, Unity 3D (C#, Visual Studio 2017) was used, following the design recommendations established in previous studies [21]. In this sense, the game interface was designed while taking into account that the target audience is older people and adapted to their needs, including the minimum necessary information in a clear and concise way, which allows the older people to understand the objective of the game. Regarding user interaction, the game was designed to be used on a tablet instead of the standard keyboard and mouse to provide a more natural interaction and facilitate the acceptance by older people [22].

Two fundamental characteristics were taken into account in the design of this serious game. First, a shopping task with high ecological validity was included, that is, a day-to-day activity of older people. In this way, a greater interest and acceptance by older people should be achieved, due to the high utility of this tool in their daily lives. The concept of ecological validity is determined by the degree of representativeness, that the game is represented in a form and context that correspond to its occurrence in everyday life, as well as by the level of generalization that the results are able to explain similar tasks in everyday life [23]. Second, the difficulty level of the game was customized by professionals according to the needs of each older person, as detailed in the description of each game in the following sections.

An additional characteristic included in the design of the game was that immediate feedback is provided to the user at the end of each game, showing the attempts, hits, and failures made after the user performance. Moreover, positive feedback with encouragement messages is showed to the user during the completion of the game, such as “Come on, you’re about to get it,” “You almost got it. Try again,” “Surely you have the name on the tip of your tongue,” and “Don’t worry. Let’s go with another product.”

### Game Description

#### First Exercise: Shopping in a Hypermarket

Episodic declarative memory is the neurocognitive function that is affected the earliest in Alzheimer disease due to the initial involvement of the hippocampal formation in the medial temporal lobe, so the stimulation of episodic declarative memory in people with MCI who are affected by mnesic impairment is of utmost importance.

This exercise is designed with the specific aim of stimulating verbal intentional episodic declarative memory at the level of the 3 mnesic processes of encoding or fixation, consolidation or storage, and recall or retrieval of information, thus consisting of 3 phases:

- *1.1 Learning Phase*: memorization of a shopping list
- *1.2 Interference Phase*: preparation of the money to pay for the purchase
- *1.3 Recall Phase*: purchase of products from the shopping list

The level of difficulty can be adjusted by the number of products on the shopping list, the number of product categories, the memorization and interference times, the number of attempts, or the total allowed time.

#### Second Exercise: Ordering Products in a Hypermarket

On the one hand, expressive language impairment manifests itself very frequently and early in the form of anomia, a naming deficit that consists of the difficulty to recall the names of objects. Due to the great frustration caused by this early expressive language difficulty for people with MCI, it is essential to include naming stimulation tasks within the global stimulation of expressive language in cognitive stimulation programs.

On the other hand, complex visual gnosias, in which the visual recognition and identification of objects is hindered by a modification of the characteristics of the objects’ images, are one of the cognitive functions that are affected in the early stages of several primary cortical degenerative dementias, hence the importance of their stimulation.

This exercise is designed with the specific aim of stimulating language at the level of naming and complex visual gnosias in 2 phases:

- *2.1 Phase 1*: hypermarket product naming
- *2.2 Phase 2*: visual recognition and identification of hypermarket products

The difficulty can be customized by adjusting the product familiarity percentage, the number of products that appears, whether or not to provide help to recognize the products, or the allowed time.

#### Third Exercise: Making Payments

Executive attentional control processes are affected early in several types of primary and secondary dementias such as subcortical vascular dementia. Working memory, a key element of executive attentional control, is the ability to maintain and manipulate information in on-going cognitive activity, such as money management for shopping. It is therefore very important to place great emphasis on the stimulation of these cognitive processes to maintain the highest possible level of functional independence of each person.

This exercise consists of 3 phases and is designed with the specific aim of stimulating the working memory through calculation tasks by simulating purchase payments:

- *3.1 Phase 1*: payment of the purchase
- *3.2 Phase 2*: check the change (money returned)
- *3.3 Phase 3*: check the price of products on the purchase receipt with the catalog of offers

Professionals can adapt the difficulty by adjusting whether or not to include decimals in the amount to be paid or the change, the number of attempts, the allowed time, the number of correct banknotes or coins in the change, the number of products, pages and sections in the catalog of offers, or the rate of products with erroneous price.

#### Fourth Exercise: Organizing the Purchase

Executive functions, such as planning and organization, reasoning, cognitive flexibility, or monitoring when problem-solving, are crucial functions for a good performance of any adult in advanced and instrumental ADL. In the context of cognitive treatments for MCI, the stimulation of executive functions is a compulsory subject given their close interdependence with the maintenance of a high level of functional independence and personal autonomy in ADL.

This exercise is designed with the specific objective of stimulating this executive function of organization, as well as abstract reasoning, performance monitoring, visual gnosias, semantic memory, and visuospatial function.

The only phase of this exercise consists of the arrangement of the purchase products in the corresponding rooms of the house.

In this case, the adaptation of the difficulty can be achieved by adjusting the number of products on the list and the maximum time allowed to complete the task.

### Acceptance and Usability Study

A preliminary validation of the serious game was carried out by 4 participants from the Association of Friends of the Minimally Invasive Surgery Centre in Cáceres, Spain; 5 participants from senior centers in Castelo Branco, Portugal; and 10 participants who attended the FEHISPOR fair held in Badajoz, Spain. The nonprobability intentional sampling technique was used to conduct the recruitment of participants, with inclusion criteria of older people aged 60-80 years with MCI. Through this method, older people from those attending various demonstrations of the game at the aforementioned events and venues were recruited to voluntarily participate in the study.

Participants tested the game until all exercises were completed and then filled in a questionnaire. The questionnaire consisted of questions scored on a 5-value Likert scale (1=lowest value, 5=highest value) about the acceptance and usability of the game, assessing (1) the user’s perception and degree of satisfaction when using the game and (2) the design and layout of the game. A descriptive analysis with the average values of the answers provided by the participants was performed.

### Ethical Considerations

Both ethical approval and written participants consent were waived for this study because they were not within the scope of Law 14/2007 of 3rd July on Biomedical Research, due to this study not involving any invasive procedure on the participants. Only verbal informed consent was considered sufficient. All data have been deidentified, and there was no compensation for participation.

## Results

### Game Implementation

#### First Exercise: Shopping in a Hypermarket

This exercise consists of 3 phases.

**Figure 1.**
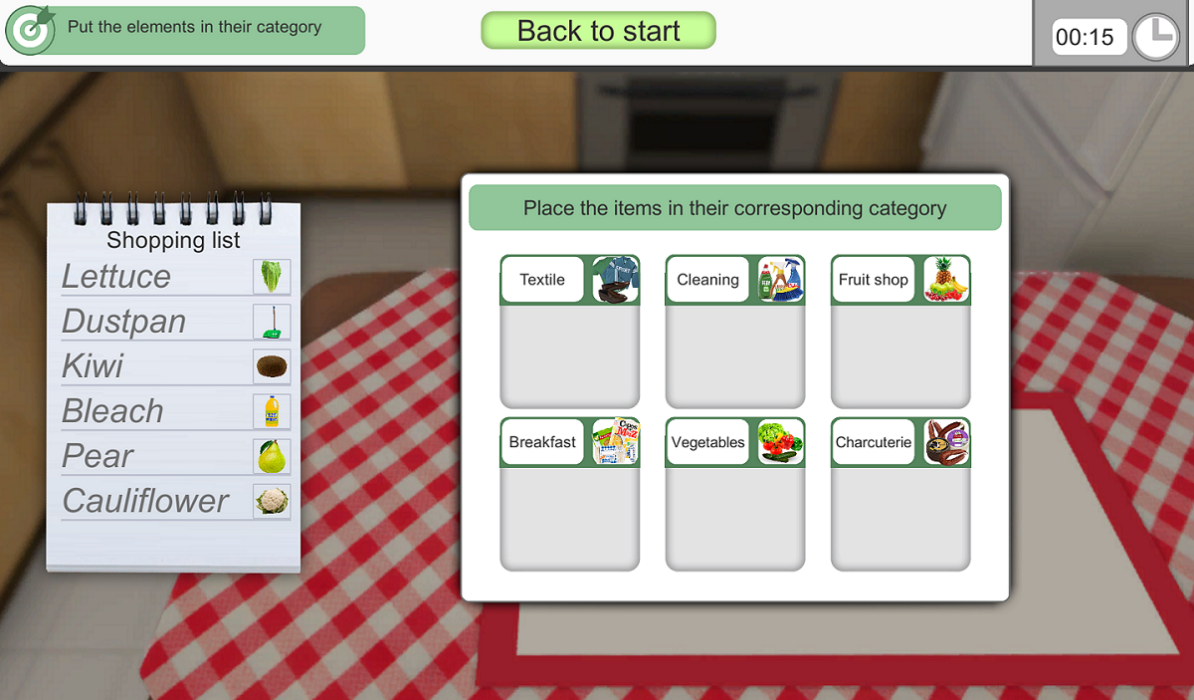
The first exercise stimulates episodic declarative memory with 3 mnesic processes: encoding, consolidation, and recall. The user must learn a series of products from a shopping list.

In *1.1 Learning Phase*, the user must learn a series of products from a shopping list in 3 subphases (Figure 1): (1) organization of the list by categorization, since the structure of the information facilitates its deep encoding; (2) identification of specific characteristics that differentiate the products, since better fixation of information with clues optimizes recall; and (3) intentional memorization of the list in a specific time frame.

In *1.2 Interference Phase*, the task simulates the preparation of the wallet with the money to pay for the purchase. It activates the processes of attentional control at the level of selective attention to choose the correct banknotes or coins, working memory to sum the money, and monitoring of the execution to avoid errors.

In *1.3 Recall Phase*, the user is presented with a map of a hypermarket where the sections are marked out, and the user has to fill a shopping basket with those products from the shopping list studied in the *1.1 Learning Phase* among distracting products. The aim is to stimulate the mnesic process of recalling short-term episodic declarative memory.

#### Second Exercise: Ordering Products in a Hypermarket

This exercise consists of 2 phases.

**Figure 2.**
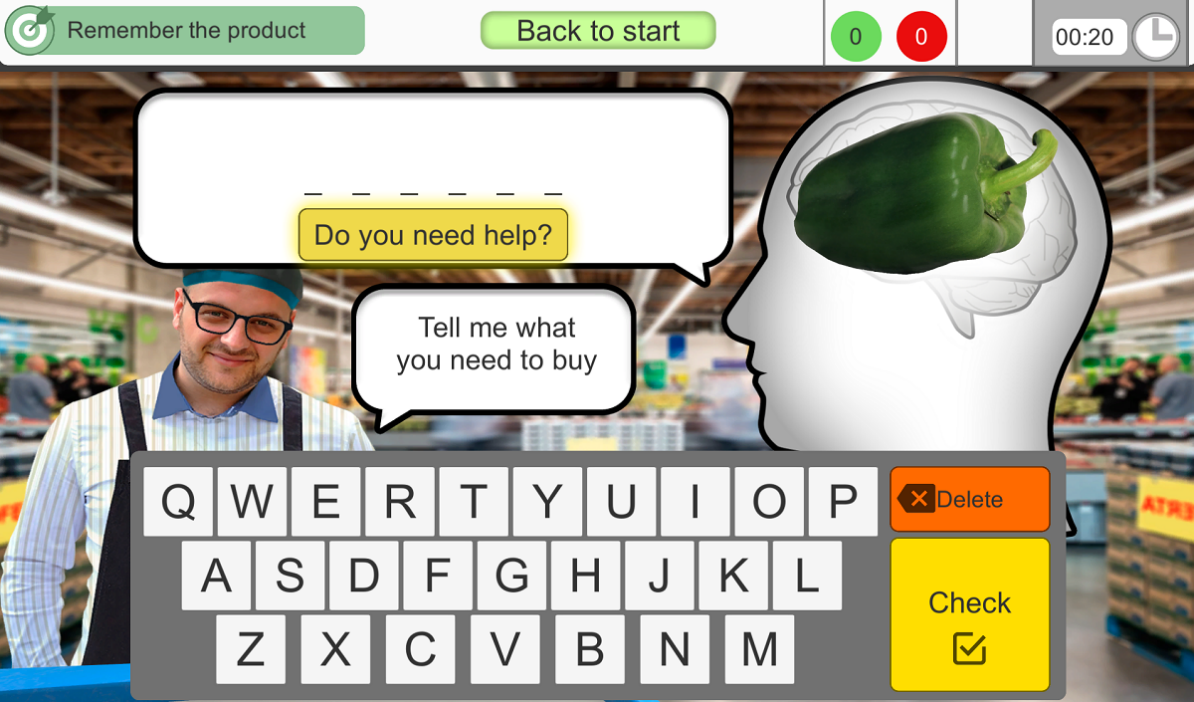
The second exercise (2.1) stimulates expressive language. The user has to write the names of the products that appear in the images.

In *2.1 Phase 1*, the user has to write the names of the hypermarket products that appear in the images (Figure 2).

**Figure 3.**
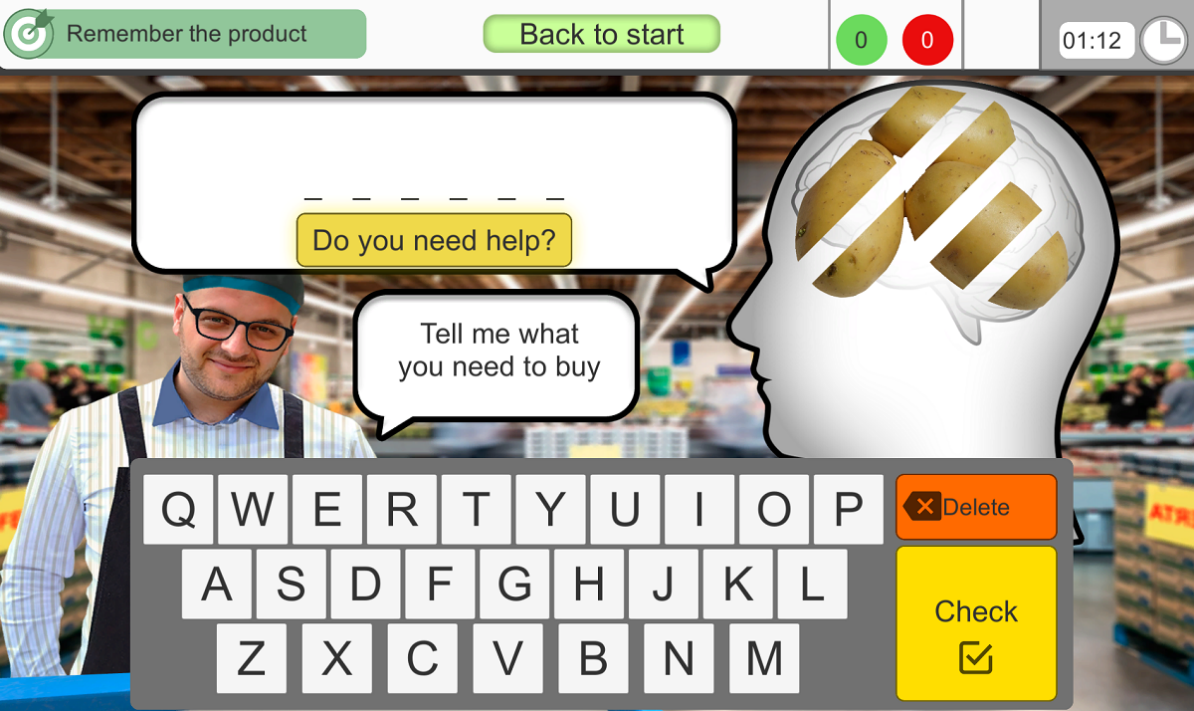
The second exercise (2.2) stimulates complex visual gnosias. The user has to recognize and write the names of the products through their distorted images.

In *2.2 Phase 2*, the user has to recognize and identify the hypermarket products through their distorted images and subsequently name these products (Figure 3). The emphasis is placed on the stimulation of complex visual gnosias, making it difficult to recognize and identify the objects to be named.

#### Third Exercise: Making Payments

This exercise consists of 3 phases.

**Figure 4.**
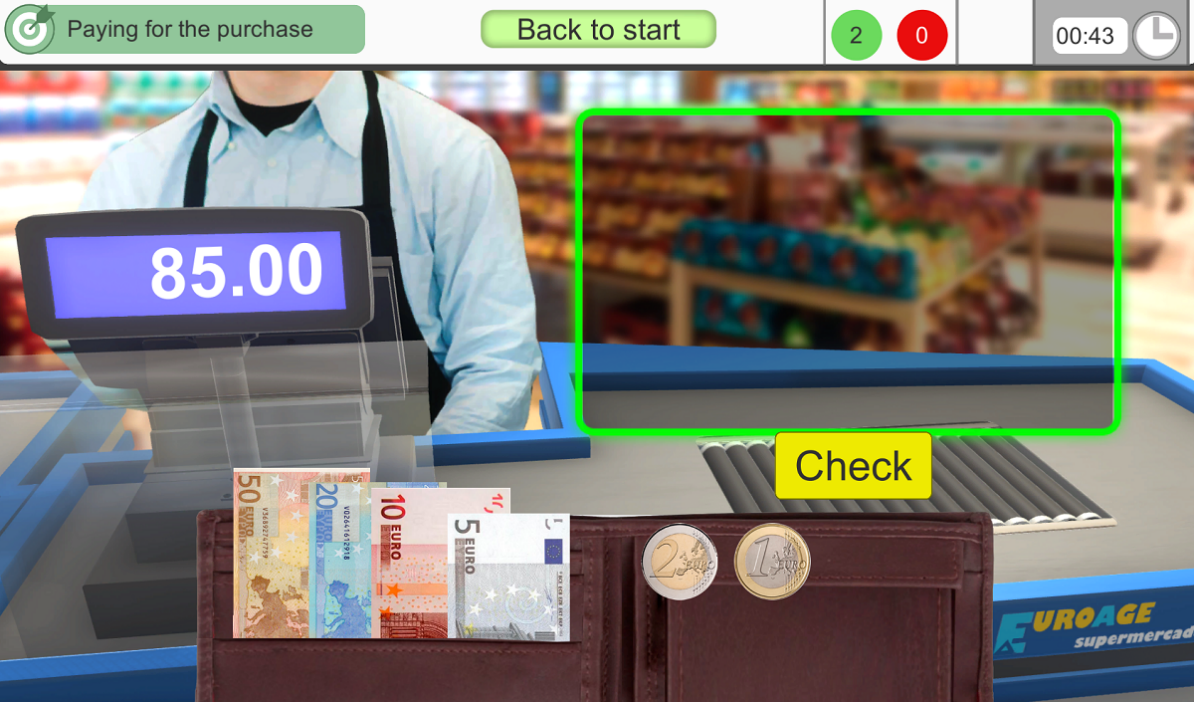
The third exercise stimulates executive attentional control. The user has to use banknotes or coins to make the exact payment of the purchase.

In *3.1 Phase 1*, the user has to use banknotes or coins to make the exact payment of the purchase (Figure 4).

In *3.2 Phase 2*, the user must check whether the change (money returned) received is correct, and if not, he or she must select the banknotes or coins necessary to make the change amount correct.

In *3.3 Phase 3*, the user has to check the price charged for each product on the purchase receipt to verify whether it corresponds to the price for that product in the catalog, and in the case of error, to mark on the receipt the products for which the amount on the receipt is erroneous.

#### Fourth Exercise: Organizing the Purchase

**Figure 5.**
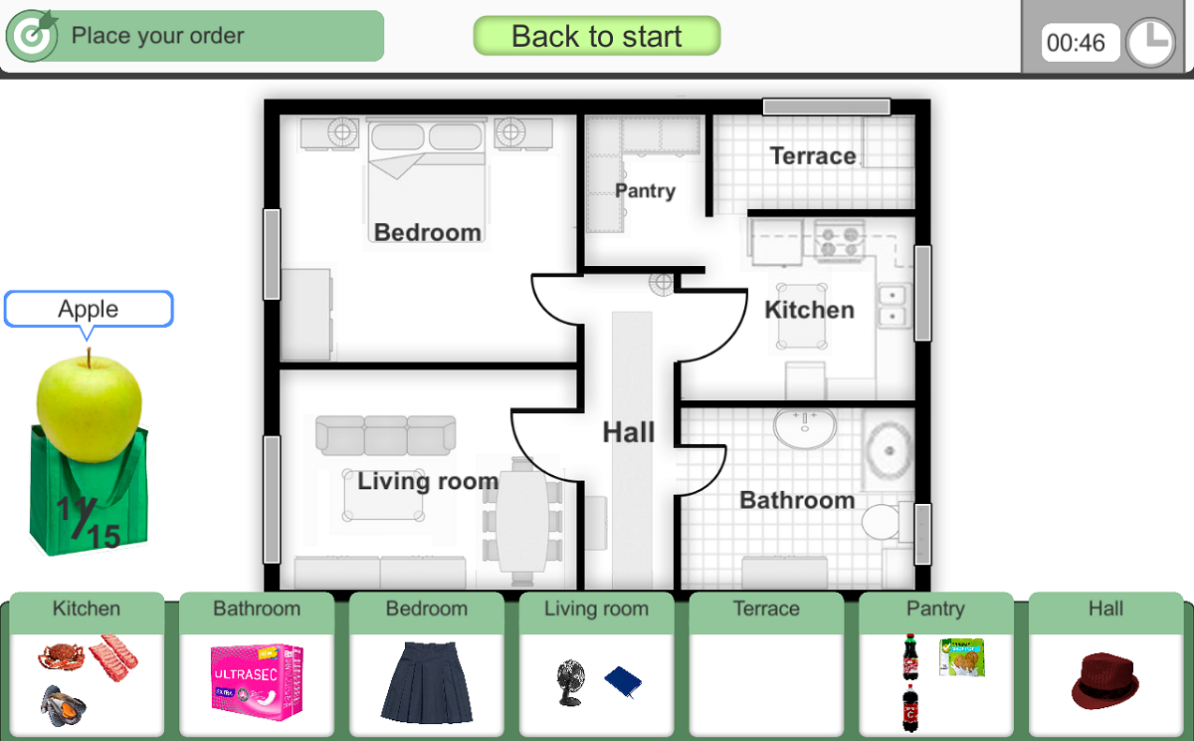
The fourth exercise stimulates executive functions. The user must arrange the products of a purchase into the rooms of a house.

In this exercise, the user must arrange the products of a purchase into the rooms of a house, whose floor plan appears on the screen and consists of the following spaces: kitchen, terrace, bathroom, pantry, living room, and bedroom (Figure 5). Within each of these house spaces, the correct storage location is flexible according to each product.

### Acceptance and Usability

**Figure 6.**
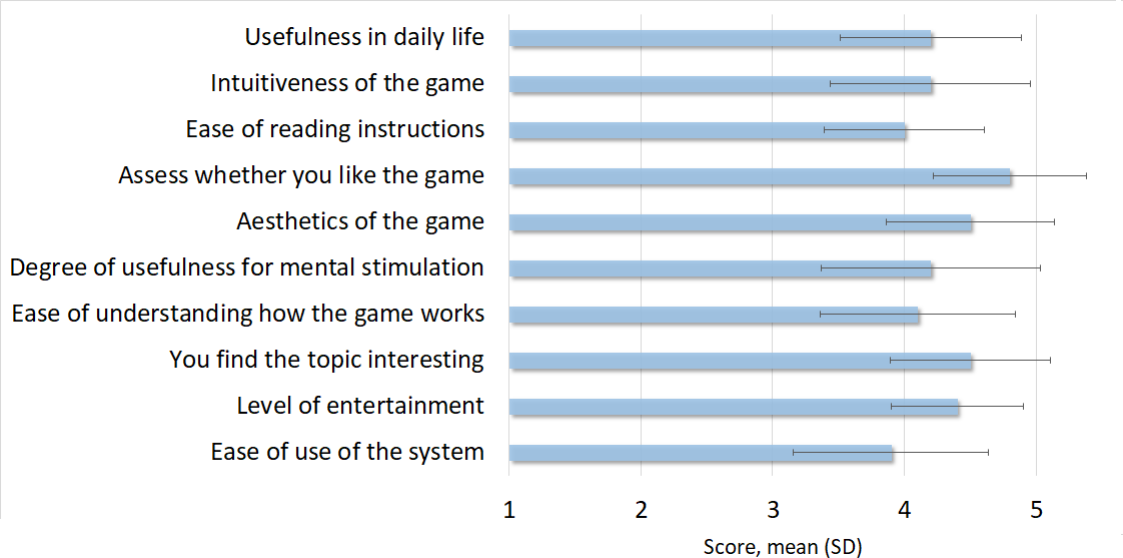
Scores of older people who participated in the validation.

A total of 19 people participated in the study (Table 1) and provided their opinions regarding the serious game, which are shown in Figure 6.

**Table 1.** Demographic characteristics of participants.

Characteristics		Value (N=19)
Age (years), mean (SD)		75.3 (1.4)
**Gender, n (%)**		
	Woman	16 (84)
	Man	3 (16)
**Level of education, n (%)**		
	Secondary (high school)	19 (100)
**Smartphone experience (frequency of use), n (%)**		
	Once a week	19 (100)

All items were highly valued with a score over 3.5 out of 5, which is the threshold for an item to be considered as positively validated, and the average value was 4.28 (SD 0.67). On the one hand, the most valued aspects were that participants like the game (mean 4.8, SD 0.58), both from the aesthetics point of view (mean 4.5, SD 0.64) and the interesting topic (mean 4.5, SD 0.61) of the game. On the other hand, the items that had the lowest score were the ease of use of the system (mean 3.9, SD 0.74) and the ease of reading the instructions (mean 4.0, SD 0.61).

## Discussion

### Principal Findings

From a cognitive point of view, the first effects of deterioration in older adults are directly related to the loss of the ability to carry out some instrumental ADL. However, there are multiple studies showing that people who read or play games are less likely to develop dementia or even Alzheimer disease [24,25]. In particular, the use of serious games has proven its value as a cognitive therapy for older people [26]. In the literature, different games have been designed and validated for cognitive stimulation [17,18], but it is not common for these games to use tasks focused on ADL. The aim of this work has been to describe how a serious game has been designed and implemented for cognitive stimulation of older adults by means of memory, naming, calculation, and organization exercises, which are key in ADL such as shopping. In this way, this serious game deals with the most frequent cognitive problems of older people indicated by health professionals.

According to the literature, it is not completely clear to what extent existing health care research explicitly distinguishes between gamification and serious games [27]. Serious games refer to the use of games and gaming technology for purposes other than just entertainment or fun, including health purposes. They can have direct or indirect positive physiological and psychological effects on people, which is precisely the objective of serious games in health and health care [28]. Our work applies to this definition because our serious game was designed for cognitive stimulation of older people with MCI and could have direct positive psychological effects on older people for the prevention or treatment of such cognitive impairment.

The preliminary pilot study carried out to validate the usability of the serious game showed that participants had a great opinion of this game and considered the theme interesting, entertaining, and useful for mental stimulation, so we can think a priori that the game could be well accepted among older people. Users found the serious game intuitive and aesthetically appealing; therefore, it meets the principles of simplicity and intuitiveness for the design of user interfaces for older people to avoid extracognitive load for the user [29].

Taking into account preferences of older people, game themes should meet their interest because they have a predilection for games related to real life [30]. In this sense, ecological validity was considered in the design of our serious game, since it is important for the validation of cognitive skills that influence functional tasks in real-world contexts [31]. Moreover, the fact that the difficulty of the game can be set by the professional to provide an achievable difficulty for each older adult user is important to motivate them to play and avoid frustration, anxiety, or negative emotions when playing [30].

Previous studies have described the benefits of using tablets for cognitive stimulation [32], but at the same time, rejection and barriers for older people in the use of these technologies have also been described [33]. In this regard, the results obtained in this study indicate that the game is easy to use and understand. However, these are preliminary findings as the study participants use smartphones in their daily live, which greatly reduces the rejection of this type of technology. Nevertheless, as future work, it is necessary to improve the interaction and facilitate the use of the game to avoid the rejection by older people, since results obtained from the questionnaires regarding the ease of use and instructions of the game were positive, but there was room for improvement.

In relation to the use of technologies by older adults and to digital health, the European Commission encourages improving the digitalization of health systems to fight health inequalities [34]. However, digital health services and devices are useless if consumers, in this case older people, do not have the skills or understanding to use them. For that, in the digital age, more than ever, literacy in digital health is a critical first step to improve the quality of life.

A feature of this serious game is that it provides feedback to the user at the end of each stage of the game, indicating whether the task was correctly completed or not and the type of mistake that was made. This agrees with Brox et al [35], who stated that when older people achieve their goal, feedback should be immediately provided. Moreover, to avoid frustration, which is another key aspect of the game, the system also provides encouraging messages during the completion of the game, so that the user can try again. This positive feedback for encouragement favors a successful experience of older user with the game [36], allowing them to achieve the goal with high motivation [30]. The use of narratives and the low complexity of the game are factors that motivate older people to play [37,38]. In this way, motivational mechanisms have been included in this serious game, which is the fundamental element for success of new training exercises.

According to the literature [39], game design features related to the game genre (GG), game nature (GN), and game development strategy (GDS) are necessary to develop a serious game. The GG covers sports, simulation, and strategy, among others. GN features include player perspective taking (first-person perspective vs third-person perspective), gameplay mode (multiplayer vs single player), type of scenery or in-game environment (realistic, fantasy themed, or simple), the presence or absence of playable characters, and the level of immersion applied to the use of immersive or nonimmersive virtual reality. GDS refers to custom-made serious games developed specifically for the study in question or direct-to-consumer approaches. In our work, regarding the GG, we can associate gender to the simulation of ADL, such as shopping, thus meeting this design criterion. In the case of GN, a first-person perspective, single-player experience, realistic scenario, the absence of playable characters, and no immersion was implemented. According to these design features, those of interest that have not been included in our serious game and could be implemented and tested in the future works are as follows: (1) a multiplayer gameplay mode: in this way, older people could simulate going shopping together with their loved one, family member, or friend, which is something they are probably used to in their life, thus increasing the feeling of sociability; and (2) changing the level of immersion to include the use of virtual reality: the use of virtual reality could be of interest, so that the older person is immersed in the game and can feel as if they are actually doing the shopping task, especially in the exercises that involve going to the supermarket, such as asking the shop assistant for the products, moving through the sections of the supermarket and filling the shopping basket with the products on the list, or even arriving home with the shopping basket and moving through the different sections of the house to place the products. All of these features would involve older people in a more realistic environment. However, the effort (human and material resources) involved in implementing these features, as well as the acceptance of older people to the use of this type of virtual reality technologies, either with glasses or simulating on a PC with a keyboard and mouse, would have to be carefully studied. Finally, regarding the third game design feature of GDS, our serious game has been implemented solely for our study, but it will be freely available for download and use by older people and health care institutions who wish to use it.

This serious game presents an alternative format to traditional interventions for older people, so we suggest defining a protocol to assess the effects on the cognitive function of older adults with a long-term intervention after a period of time to test the effectiveness of this serious game.

### Limitations

The study has the limitation that the pilot study used a small sample size belonging to a limited geographical area, which may influence the generalizability of results. Therefore, it is necessary to extend these results with a larger sample size, including people of different cultural levels, with different experiences in the use of tactile devices, as well as people with different levels of cognitive impairment, to test the robustness of our findings.

### Comparison With Prior Work

Ecological validity has been taken into account in the design of the serious game in this work, with tasks focused on ADL such as shopping. This is not common in the different games designed and validated for cognitive stimulation found in the literature, some of which focused on sport simulations, quizzes with text and images, music tools, arithmetic and reading calculations, etc [17], whereas others use a fantasy world setting, minigames, puzzles, or scenarios to cope with stressful or negative situations [18]. The innovative factor of including ecological scenarios of ADL tasks favors a greater interest and acceptance by older people and could help to assess the cognitive status to prevent or control the progress of the cognitive impairment.

### Conclusions

In this work, an innovative serious game for cognitive stimulation of older people has been designed and developed, focusing on the ADL of shopping, incorporating motivational elements, and allowing for difficulty adaptability. The set of exercises included in the serious game have been described, including the theoretical basis on which each exercise has been implemented, which deal with the most frequent cognitive problems of older people associated with episodic declarative memory, naming, calculation, and organization. This study could serve as a basis for future serious games for cognitive stimulation of older people that may benefit from the knowledge obtained about the design strategies followed.

A pilot usability study carried out with older adults has shown that this serious game is intuitive, provides a high level of entertainment, and is useful for its application in daily life. In conclusion, according to the preliminary results obtained, we think that the serious game could be widely accepted by older people and, therefore, could become a tool that contributes to delaying their deterioration and increasing their independence. In this way, the serious game developed could contribute to increase the low degree of awareness of older people regarding the importance of leading an active life.

## Abbreviations

ADL: activities of daily living
GDS: game development strategy
GG: game genre
GN: game nature
ICT: information and communication technology
MCI: mild cognitive impairment
